# Personality Associations With Smartphone and Internet Use Disorder: A Comparison Study Including Links to Impulsivity and Social Anxiety

**DOI:** 10.3389/fpubh.2019.00127

**Published:** 2019-06-11

**Authors:** Jessica Peterka-Bonetta, Cornelia Sindermann, Jon D. Elhai, Christian Montag

**Affiliations:** ^1^Molecular Psychology, Institute of Psychology and Education, Ulm University, Ulm, Germany; ^2^Department of Psychology, University of Toledo, Toledo, OH, United States; ^3^Department of Psychiatry, University of Toledo, Toledo, OH, United States

**Keywords:** internet use disorder, smartphone use disorder, internet addiction, personality, smartphone addiction, big five model of personality, problematic internet use, problematic smartphone use

## Abstract

The present work aims to replicate findings linking specific personality traits with Internet and Smartphone Use Disorder (IUD/SUD). Specifically, earlier research demonstrated that tendencies toward IUD and SUD are associated with high Neuroticism and both low Conscientiousness and low Agreeableness, while IUD (but not SUD) tendencies are negatively related to Extraversion and SUD (but not IUD) tendencies are negatively associated with Openness (1). In the aftermath of the replication crisis in psychology and related disciplines, it has become increasingly important to replicate findings in psychological research. Therefore, we revisited this earlier study by investigating (i) a sample from different countries and (ii) using different questionnaires to assess IUD, SUD and the Five Factor Model of Personality than the earlier work by Lachmann et al. (1). By applying such a design, we believe that replicating results from this earlier study hints toward generalizable associations being (largely) independent from that sample's specific cultural background and instrumentation. Importantly (iii) we used a larger sample consisting of *N* = 773 in the present study to have higher statistical power to observe the initially reported associations. Additionally, we investigated the role of impulsivity and social anxiety on IUD/SUD, further illuminating the nature of these potential new disorders. Indeed, we were able to reaffirm the aforementioned correlation patterns between personality and IUD/SUD in the present work to a large extent, with low Conscientiousness and high Neuroticism being most robustly associated with higher IUD/SUD. Furthermore, social anxiety and impulsivity showed positive correlations with IUD and SUD, as expected.

## 1. Introduction

According to the latest estimates, 2.71 billion people use a smartphone worldwide (2). This number is remarkable, because the modern smartphone has existed only since the iPhone's introduction (in January 2007). Without a doubt, the smartphone in general represents one of the most relevant accelerators toward a digitally connected world (3). With the smartphone in nearly everyone's pocket, it is of high relevance to also investigate the smartphone's impact on interpersonal communication patterns, productivity and other domains of everyday life. The authors of Montag et al. (4) use objective smartphone tracking methods, finding that typical users spend approximately 2.5 h on their phones, with the largest time spent on social media applications.

We believe that it is important to not over-pathologize everyday life behavior (5) by acknowledging the positive effects smartphones can have on our lives aside from the often mentioned negative effects. If used in the right manner, smartphones enable meaningful social communication via far distances at low cost and they help to navigate unknown geographical territory. The right usage of the smartphone can make us more productive (6), but a certain kind of usage pattern, perhaps best characterized by fragmentation of everyday life, might reduce our productivity at work (7). Furthermore, if the phone is used with high frequency, it might lead to symptoms of “inattention” [(8); see also the work by (9)]). Among strangers, smartphone usage has shown to undermine smiling behavior (10) and in families it might be detrimental to paying attention to one's own children (11), negatively impact communication patterns at family meals (12) and the enjoyment of face to face interactions [(13); see also (14)]. For a general overview on smartphone usage in the realm of cognitive functions, see the review by Wilmer et al. (15).

A growing body of research aims to investigate whether excessive usage of the smartphone might resemble an addictive behavior. This topic is controversial and therefore the research community has proposed different nomenclatures such as “smartphone addiction” or “problematic smartphone use.” Of importance, no matter how this debate will ultimately be resolved, excessive use of smartphones is not included in the current versions of DSM (-5) and ICD (-11). Thus, excessive usage of the smartphone is not officially recognized as a disorder, currently. However, some of the aforementioned literature already stressed that maladaptive usage of smartphones can be detrimental to personal and work life. In fact, several new research studies highlight relationships between “problematic smartphone use”, anxiety, depression (16, 17) and negative affect in general (14, 18). Samaha and Hawi (19) reported that stress mediates between “smartphone addiction” and life satisfaction, shedding more light on the general topic investigating links between Internet Use Disorder (IUD)/Smartphone Use Disorder (SUD) and life satisfaction. Lachmann et al. (20) replicated the correlation between high IUD/SUD and low life satisfaction but also found that high IUD and SUD are linked to low empathy. In the present paper, we will use the term “Smartphone Use Disorder” (SUD). We use this term for several reasons: Excessive use of the smartphone and Internet overlap considerably [about *r* = 0.50, (20)] and one of the most prominent process models to explain the development of excessive Internet usage introduced the term “Internet Use Disorder” (21). From our perspective it is meaningful to use this term, because it points to the idea that a person does not have problems due to the Internet *per se*, but because of using the Internet in a certain way. Moreover, the term “disorder” has been coined in reaction to including Gaming Disorder, a specific form of IUD, in the latest version of ICD-11. Of note, usage of the term “disorder” in the present work aims at finding a common nomenclature in the area of (online) addictive behaviors instead of being a final judgment on the actual nature of this still new phenomenon. In the present paper, we chose not to use the term “problematic smartphone use,” because it either could describe a person transitioning to or already at a psychopathological state of SUD. Of course, all current nomenclatures in this area come with difficulties [see also (22)].

Whereas IUD might be the overarching umbrella term of the research discipline attempting to understand excessive Internet use behavior, SUD might belong to a sub-division of so-called mobile IUDs. Ubiquitous access to the Internet, from anywhere a phone signal is available, might be one of the key drivers of habit formation in the context of smartphone usage with its most extreme form perhaps being a Use Disorder (23). A study conducted by Rumpf et al. (24) in Germany demonstrated that 1.5% of the population where suffering from IUD, whereas Müller et al. (25) found that 2.1% of their German sample could be characterized as addicted to computer games. A meta-analysis conducted by Cheng and Li (26) reported prevalence rates of IUD between 2.6% (Northern and Western Europe) and 10.6% (Middle East). Representative prevalence reports for SUD do not yet exist to our knowledge.

Of note, a prominent model to understand IUD (or “problematic Internet use”) was developed by Davis (27) distinguishing between generalized and specific forms of IUD. Whereas generalized IUD describes excessive usage of diverse online content, specific forms of IUD are characterized by more or less exclusively overusing one online area such as gaming, pornography or Internet communication platforms/social media. Most of the prominent measures to assess tendencies toward IUD and SUD query the respondent about detrimental effects of their online usage on areas such as sleep, interpersonal relations, but also classic addiction symptoms such as preoccupation and withdrawal (28, 29). However, terms such as “smartphone” or “online” in the respective inventories might lead to the activation of different concepts in participants' minds while responding to such an “unspecified” questionnaire (30).

According to the I-PACE model by Brand et al. (21), IUDs develop within a complex interaction of Person, Affect, Cognition and Executive variables. For the present work, we stress in particular the Person variables. Here, Brand et al. (21) describe the relevance of personality traits such as low conscientiousness and (a history of) psychopathology to identify persons with vulnerability to develop IUD. Regarding the prominent Big Five Model of Personality, a review observed that high Neuroticism and low Conscientiousness might be among the most relevant variables to predict IUD (31). Neuroticism itself is a well-known risk factor for depression (32) and IUD has been also associated with negative affect/depression (33, 34). Low Conscientiousness might be characterized by low will-power [see also overlap with low Self-Directedness; (35)] and within these terms explain why persons have difficulties controlling their online usage. Several new studies investigated such personality associations with SUD. Research on the link between SUD and personality consistently show that Extraversion is not linked to SUD, but high Neuroticism is associated with SUD (1, 36–38). The results involving the other personality traits, Openness, Agreeableness, and Conscientiousness, on the other hand are inconclusive. Agreeableness was not significantly associated with SUD in Roberts et al. (37) nor Hussain et al. (38), but low Agreeableness was significant in the sample used by Lachmann et al. (1). Finally, Openness and Conscientiousness were not significantly associated with SUD in Roberts et al. (37), but low Openness and low Conscientiousness were linked to SUD in Lachmann et al. (1) and Hussain et al. (38).

Comparably few studies investigated relationships between IUD and SUD, in particular when also taking into account personality associations. Past research led by Kwon et al. (29) on the development of the Smartphone Addiction Scale (SAS) has uncovered partial overlap between IUD and SUD. A study by Lachmann et al. (1) investigated whether the same personality traits could be linked to overuse of both platforms. Their results suggest that a common personality structure linked to both IUD and SUD indeed exists, which explains the overlap. In fact, both higher IUD and SUD scores were associated with higher Neuroticism, lower Agreeableness and lower Conscientiousness. Besides the similarities between personality traits, IUD and SUD, the authors have also found differences. Whereas lower Extraversion is associated with higher IUD scores, Extraversion was unrelated to SUD. Furthermore, low Openness was linked to high SUD scores, but was unrelated to IUD. Besides personality related differences between mobile and non-mobile Internet Use Disorder (both under the umbrella term IUD), there is also noteworthy conceptual differences. Online content is constantly available on mobile, which is not true for desktop computers. This led to the development of different services which swapped from the mobile to non-mobile devices and the other way round. E.g., in 2004 Facebook was founded and started in the pre-era of the smartphone as a desktop application. Now different forms of this product are available on smartphones. The WhatsApp application represents an example for the development of a tool switching from mobile to non-mobile. In sum, availability and applications differ between mobile and non-mobile Internet.

Since the original study (1) was conducted on a German sample, the question arises whether similar results can be found with a sample from a different cultural background. Additionally, it is unknown if the observed correlations between IUD, SUD and personality depend on the specific instruments used to assess the three constructs. The goal of the present work is thus to replicate findings on a different sample using different questionnaires. Assuming that similar results could be observed regardless of the self-report methods used, successful replication would be an indication that the prior results are valid and not dependent on specific measurement methods (nor on the cultural background of the sample). The inventories we chose to assess IUD, SUD and personality, had to comply with two criteria: First, they had to possess good psychometric qualities and second, they had to be brief in length. The latter was important to keep the survey as short as possible and ultimately motivate as many participants as possible to complete the survey. In Lachmann et al. (1), IUD was measured with the German version of the Internet Addiction Test (IAT) by Young (28) as used in Montag et al. (39) and comprising of 20 items, whereas in the present study, we used the short version of the IAT (s-IAT) by Pawlikowski et al. (40) comprising of 12 items. Because the s-IAT was derived from the IAT, not only does s-IAT measure the same concept as IAT but it also consists of a subset of the original IAT items. Lachmann et al. (1) used the Smartphone Addiction Scale (SAS) published by Kwon et al. (29) to measure SUD, while we used the Smartphone Addiction Inventory (SPAI) from Lin et al. (41). Whereas the SAS was developed using the Korean self-diagnostic program for IUD and consists of 33 items, the SPAI consists of 26 items and is based on the Chinese Internet Addiction Scale and phantom vibration and ringing syndrome questionnaire. The SAS demonstrates a six factor structure consisting of daily-life disturbance, positive anticipation, withdrawal, cyberspace-oriented relationship, overuse and tolerance, whereas the SPAI consists of four factors—compulsive behavior, functional impairment, withdrawal and tolerance. Beyond obvious overlap in the withdrawal and tolerance factors, both SAS and SPAI also have similar items like “Feeling tired and lacking adequate sleep due to excessive smartphone use” (SAS) and “I feel tired on daytime due to late-night use of smartphone.” (SPAI). Investigating in detail the differences and similarities between both scales would be beyond the scope of this work, nonetheless it is worth acknowledging that generally, these scales differ in the specific items they use but are overall similar in content, which is to be expected because they intend to assess the same concept. Regarding personality measurement, in Lachmann et al. (1) the NEO Five-Factor Inventory (NEO-FFI) by Costa and McCrae (42) was used [German version by (43)], while we assessed personality using a short version of the Trait Self-Description Inventory [TSDI, (44)] as proposed by Olaru et al. (45). Both instruments are based on the same theory and ultimately factors of personality. The short form of the TSDI used in this work was developed with items from the NEO-FFI, among others. Consequently, some items from the short form of the TSDI can be found in the NEO-FFI as well, but there are also non-overlapping items. Whereas the short form of the TSDI used in this study consists of 42 items, the NEO-FFI used by Lachmann et al. (1) comprises 60 items. Summing up, the scales chosen in this research were shorter than the questionnaires used in Lachmann et al. (1) and could thus be completed faster, but considering the way they were developed and the items they comprise, we assume that they are equivalent to the questionnaires used in Lachmann et al. (1).

Unlike in the original study, we did not assess Self-Directedness and results reported below will thus not mention this construct (please note that cross-cultural effects on low Self-Directedness and higher IUD have been robustly observed earlier by Sariyska et al. (46)—due to economic reasons we did not revisit this topic again).

In addition to associations with the Big Five of Personality, the present work also investigates putative links with other relevant person variables known to be involved in the development and maintenance of IUD according to the I-PACE model, namely (high) impulsivity and (high) social anxiety. Past research has revealed a positive correlation between impulsivity and IUD. In the study by Cao et al. (47), the IUD group had significantly higher scores on the Barratt Impulsiveness Scale 11 (BIS-11) subscales of attentional and motor impulsivity than the control group. In another work by Mottram and Fleming (48), the lack of perseverance (considered an aspect of impulsivity) was a significant predictor of IUD and Kwon (49) demonstrated that adolescents suffering from IUD were more impulsive than their counterparts not suffering from IUD. In this context also a meta-analysis on impulsivity and IUD on data from Taiwan is of relevance (50). For our present research endeavor, it is noteworthy that studies investigating the relationship between impulsivity and IUD, as mentioned in this text, have either been conducted many years ago or examined a specific facet of IUD such as Gaming Disorder. Given the dramatic changes in the way the Internet developed in the past few years (from a rather static, “readable” web 1.0 to a participatory, “writable,” social web 2.0), we believe it is important to revisit the link between impulsivity and IUD from time to time. Moreover, the associations between impulsivity and SUD have been less studied, but again with a positive correlation between impulsivity and SUD (51–53). It also needs to be mentioned that the latter studies were conducted in Asian countries, leaving it unclear whether the results can be transferred to Western samples.

As mentioned above, the present study also aims to illuminate the role of social anxiety as a possible risk factor for IUD and SUD. A study by Kim et al. (54) has shown that a higher preference for online interaction is linked to higher IUD scores and a more recent meta-analysis by Prizant-Passal et al. (55) confirms the positive relationship between social anxiety and IUD. With respect to SUD and social anxiety, Enez Darcin et al. (56) showed that higher scores on the Brief Social Phobia Scale (BSPS) were associated with higher scores on all subscales of the Smartphone Addiction Scale (SAS). Other work has also found positive associations between social anxiety and SAS (18, 57). Although some first evidence suggest a link between social anxiety and SUD, it largely remains unknown whether social anxiety acts as a risk factor for SUD in the same way it does for IUD.

Summing up, in the present work we expect to find both IUD and SUD associated with high Neuroticism, low Conscientiousness and low Agreeableness, while Extraversion is expected to be negatively linked to IUD but not SUD. Openness should be negatively correlated with SUD but unrelated to IUD. These associations have been observed in an earlier study by Lachmann et al. (1) in a German sample and should be replicated in this study. Similar to the original study by Lachmann et al. (1), we assume that correlation patterns between personality and IUD/SUD are to a large extent similar across gender. Given the above reviewed work, we further expect to find a positive correlation between impulsivity and IUD/SUD as well as between social anxiety and IUD/SUD. The study by Lachmann et al. (1) will be referred to as the “original study” in subsequent sections.

## 2. Methods

### 2.1. Participants

After removing outliers, the sample used in this work consists of 773 participants (*N* males = 303, *N* females = 470; see Results section for more details on outliers). These participants were recruited via an online-platform, where all questionnaires were presented in English (see the Questionnaires section for more detail). The study received approval from the local ethics committee at Ulm University in Ulm, Germany. As an incentive, the participants were offered feedback about their “Internet addiction” score, their personality as well as their social media usage. Participants came from 59 different countries altogether, while the vast majority of 580 participants stemmed from an English speaking country (USA: N = 442, UK: N = 58, Canada: N = 53, Australia: N = 15, New Zealand: N = 12). The mean age of the sample was 23.11 years (*SD* = 7.32). The online questionnaire gathered data about personality, SUD, IUD, impulsivity, social interaction anxiety, WhatsApp usage as well as Twitter and Instagram usernames. WhatsApp and social media data were not used in the present work, because they belong to a different research project.

### 2.2. Questionnaires

The short form of the TSDI (58) as proposed by Olaru et al. (45) comprising of 42 items was administered to assess personality. In the present version, a 5-point Likert scale was used ranging from *strongly disagree* (1) to *strongly agree* (5). Scores are computed for each factor separately so that every participant obtains a score for each personality dimension called Openness, Conscientiousness, Extraversion, Agreeableness, and Neuroticism. Internal consistencies in the present sample were high to very high (Openness: α = 0.79, Conscientiousness: α = 0.83, Agreeableness: α = 0.85, Extraversion: α = 0.83, Neuroticism: α = 0.87).

IUD was assessed using the short form of the Internet Addiction Test (40), which consists of 12 items and builds on a two-factor model (loss of control/time management and craving/social problems). Exemplary items are “How often do you lose sleep due to being online late at night?” (loss of control/time management) and “How often do you choose to spend more time on-line over going out with others?” (craving/social problems). Participants answered the inventory in the present work on a 6-point Likert scale ranging from *does not apply* (0) to *always* (5). An overall IAT score was computed by summing the 12 items, as well as individual scores for both factors by summing items belonging to each subscale, respectively. Higher scores indicate higher addictive tendencies toward the Internet. Total s-IAT scores ranged in our work between 0 and 60. The overall internal consistency was high with α = 0.84 (s-IAT Control α = 0.79, s-IAT Craving α = 0.79).

SUD was measured with the Smartphone Addiction Inventory (41), which consists of 26 items on a 4-point Likert scale ranging from *strongly disagree* (1) to *strongly agree* (4) and the SPAI score is a sum of all items, so that the total score ranges between 26 and 104. Exemplary items are “Although using smartphone has brought negative effects on my interpersonal relationships, the amount of time spent on the Internet remains unreduced.” (subscale compulsive behavior), “I feel aches and soreness in the back or eye discomforts due to excessive smartphone use” (subscale functional impairment), “I feel restless and irritable when the smartphone is unavailable” (subscale withdrawal) and “I find that I have been hooking on smartphone longer and longer” (subscale tolerance). Internal consistency of the overall scale was very high (α = 0.95) while internal consistency of the subscales was high to very high (SPAI Compulsive α = 0.86, SPAI Functional α = 0.86, SPAI Tolerance α = 0.76, SPAI Withdrawal α = 0.85).

Impulsivity was measured using the short form of the Barratt Impulsiveness Scale 15 (BIS-15) by Spinella (59) [see also (60) and the more recent work by (61) for information on the original scale]. The 15 items are rated on a 4-point Likert scale ranging from *rarely/never* (1) to *almost always/always* (4). The BIS-15 consists of three subscales, including non-planning impulsivity (BIS-15 NP), motor impulsivity (BIS-15 M) and attentional impulsivity (BIS-15 A) with items such as “I act on impulse” (BIS-15 M), “I plan for the future” (inverted, BIS-15 NP) and “I don't pay attention” (BIS-15 A). An overall score as well as individual scores for each subscale can be computed by summing respective items. Higher scores indicate higher trait impulsivity. Cronbach's α for the total scale as well as for the single subscale was high (BIS-15 α = 0.86; BIS-15 A α = 0.78; BIS-15 M α = 0.78; BIS-15 NP α = 0.82).

The Interaction Anxiousness Scale (IAS) by Leary (62) was used to quantify social anxiety in the present work. The questionnaire consists of 15 items ranging on a 5-point Likert scale from *not at all characteristic of me* (1) to *extremely characteristic of me* (5). A total sum of all items can be computed and higher scores indicates higher tendencies to experience subjective anxiety in social situations. The internal consistency of the IAS was very good with a Cronbach's α of 0.87.

### 2.3. Data Analysis

We replicated the analysis from the original study using R (63) and the R-packages *dplyr* (64), *papaja* (65), and *psych* (66) among others.

**Figure 1:**
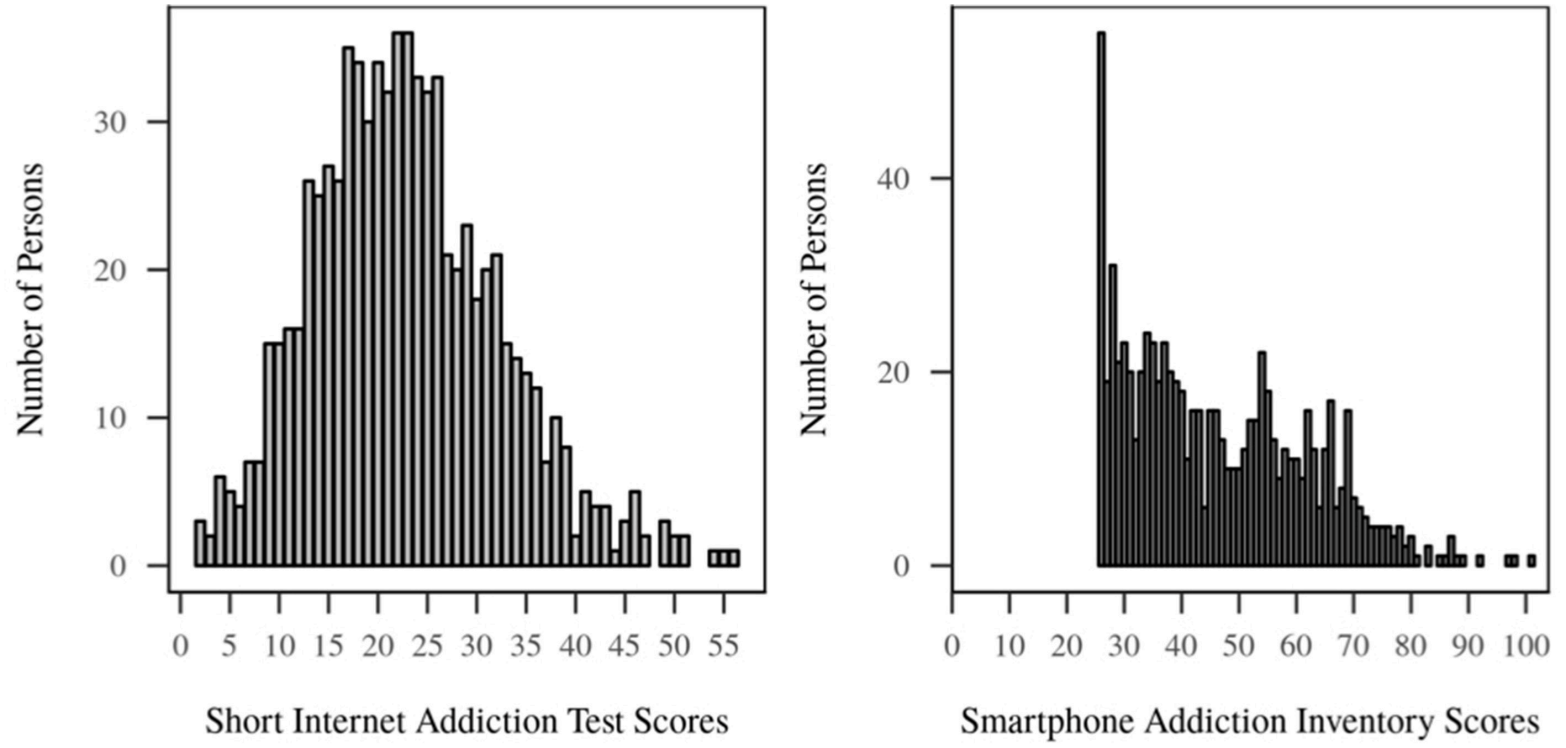
Distribution of s-IAT and SPAI scores (N = 773).

Analysis of associations between age/gender and the manifold psychological variables: We calculated correlations between age, s-IAT/SPAI, the Big Five of Personality as well as the impulsivity (BIS-15) and social anxiety measures (IAS). To account for Type I error, the correlation analysis was subject to a Holm-Bonferroni correction. In line with the original study, distributions of the s-IAT and SPAI scores were skewed (see Figure 1). In fact, none of the variables investigated in this work met the normality assumption as tested with a Shapiro-Francia normality test. For this reason, we opted for Spearman coefficients. Due to the skewed distributions of s-IAT and SPAI, we tested gender effects using non-parametric Mann-Whitney *U*-tests.

On the associations between IUD/SUD and the Big Five of Personality/impulsivity/social anxiety: These associations were computed with Spearman coefficients variables, because distributions did not meet the normality assumption. We then further compared the correlations between s-IAT and personality variables with correlations between SPAI and personality variables in order to find common personality structures to both s-IAT and SPAI as well as differences. We proceeded in the same way for BIS-15 and IAS scores by calculating correlations with s-IAT and SPAI and comparing the correlation patterns between s-IAT and SPAI. We used paired *t*-tests to test for significant differences between the correlation magnitudes. In the original study, Lachmann et al. (1) used Fisher's *z*-tests to compare correlation coefficients because of varying, non-identical sample sizes. We compared the correlation coefficients of the original study using *t*-tests but found that they were similar to the results of the *z*-tests (*z*-tests were more conservative than *t*-tests and thus the latter are overall more often significant). We further used Fisher's *z*-tests to compare correlation coefficients from the present work with those from the original study. As described in the results section we also provide the reader with models testing mediation effects when meaningful, using the function “mediate()” from the R package *psych*.

#### 2.3.1. Regression Analysis

To determine the importance of every variable on each IUD and SUD, we conducted regression analysis predicting IUD and SUD. Since this is not in the focus of the present work, the results are reported in the section 5.

Because a preliminary analysis comparing the main results between the whole sample and a subset consisting of English speaking participants did not yield any significant differences between samples, we conducted our analysis on the whole sample including both native English and non-native English speakers. Furthermore, the overall s-IAT as well as SPAI scores were used in this analysis whereas subscales were not the focus of investigation.

## 3. Results

### 3.1. Data Inspection and Outliers

A visual inspection of distributions of s-IAT and SPAI scores revealed in both cases right skewed distributions, especially for the SPAI (see Figure 1).

Eleven participants were removed from the sample for having participated twice based on their Twitter and/or Instagram usernames (note: as mentioned in the Methods section, social media data were collected but not used in this work). Another 4 participants were excluded because they reported being younger than 15 years, and an additional 17 were removed after having ticked the same answer throughout the questionnaires. After removing a total of 32 participants, the final effective sample included 773 participants.

### 3.2. Age, Gender, and s-IAT/SPAI

In the original study, gender was significantly associated with IAT, but not with SAS scores and males had higher scores on both scales. In the present study however, gender was significantly associated with SPAI (*U* = 82,342, *p* < 0.001), but not with s-IAT (*U* = 68,120.50, *p* = 0.309) and males had lower SPAI scores, but on a descriptive level rather similar s-IAT scores compared to females (s-IAT: males *M* = 23.42; *SD* = 9.74 vs. females *M* = 22.41; *SD* = 9.21; SPAI: males *M* = 44.26; *SD* = 16.59 vs. females *M* = 47.63; *SD* = 15.07).

As in the original study, age was associated with both s-IAT (*r*_*s*_ = −0.21, *p* < 0.001) and SPAI (*r*_*s*_ = -0.22, *p* < 0.001). For a complete overview of mean/median scores for all measures including male/female subsamples, see Table 1.

**Table 1:** Means, standard deviations, gender differences and correlations with age in present study vs. original study.

		**Present study**		**Original study**	
		**SPAI**	**s-IAT**	**SAS**	**IAT**
Descriptive	Male	*M* = 44.26	*M* = 23.42	*M* = 66.88	*M* = 32.45
Statistics		*SD* = 16.59	*SD* = 9.74	*SD* = 27.20	*SD* = 10.20
		*Mdn* = 39	*Mdn* = 22		
	Female	*M* = 47.63	*M* = 22.41	*M* = 64.58	*M* = 29.84
		*SD* = 15.07	*SD* = 9.21	*SD* = 23.69	*SD* = 7.83
		*Mdn* = 46.00	*Mdn* = 22.00		
	Overall	*M* = 46.31	*M* = 22.80	*M* = 65.22	*M* = 30.59
		*SD* = 15.76	*SD* = 9.43	*SD* = 24.72	*SD* = 8.66
		*Mdn* = 43	*Mdn* = 22	*Mdn* = 61.00	*Mdn* = 28.00
Gender		*U* = 82,342	*U* = 68,120.50	*U* = 31,976.00	*U* = 32,978.50
Differences		*p* < 0.001	*p* = 0.309	*p* = 0.582	*p* = 0.005
Age		*rho* = −0.22	*rho* = −0.21	*rho* = −0.16	*rho* = −0.09
Correlations		*p* < 0.001	*p* < 0.001	*p* < 0.001	*p* = 0.031

### 3.3. Personality and s-IAT/SPAI

Similar to the original study, a moderate correlation was observed between s-IAT and SPAI scores (original study: *r*_*s*_ = 0.53, *p* < 0.001, present study: *r*_*s*_ = 0.51, *p* < 0.001). The correlations between personality factors, s-IAT and SPAI can be found in Table 2. Paired *t*-tests were used to compare the correlations between personality variables and s-IAT/SPAI scores because the correlations are dependent. Fisher's *z*-tests were used to test differences in correlations between the original and present study. Similar to the original study, significantly higher negative correlations for the s-IAT compared with the SPAI scores were found for Extraversion [*t*_(770)_ = −5.08, *p* < 0.001], Agreeableness [*t*_(770)_ = −4.36, *p* < 0.001] and Conscientiousness [*t*_(770)_ = −6.01, *p* < 0.001] whereas Openness showed a significant inverse correlation with the SPAI and was not related to s-IAT scores [*t*_(770)_ = 3.65, *p* < 0.001]. Unlike in the original study, Neuroticism was correlated significantly higher with the s-IAT than with SPAI scores [*t*_(770)_ = 3.85, *p* < 0.001].

**Table 2:** Common personality relationships to short internet addiction test (s-IAT)/smartphone addiction inventory (SPAI) scores.

	**Present study**			**Original study**		
	**SPAI**	***t*_(770)_**	**s-IAT**	**SAS**	**Fisher's *z***	**IAT**
Agreeableness	−0.05	−4.36^***^	−0.20^***^	−0.11^**^	1.8^*^	−0.21^***^
Conscientiousness	−0.10	−6.01^***^	−0.30^***^	−0.23^**^	2.1^*^	−0.34^**^
Extraversion	−0.01	−5.08^***^	−0.19^***^	0.01	2.4^**^	−0.13^**^
Neuroticism	0.22^***^	3.85^***^	0.35^***^	0.21^**^	*ns*	0.26^***^
Openness	−0.13^**^	3.65^***^	0.00	−0.14^**^	−2.9^**^	−0.03
N	773		773	612		572

* *p < 0.05*,

** *p < 0.01*,

*** *p < 0.001, ns = not significant, Holm-Bonferroni corrected*.

For completeness, we also report the correlations between personality variables and s-IAT/SPAI scores for both genders separately. Males and females showed largely similar correlation patterns. Differences can be observed in the correlation between s-IAT/SPAI scores and Agreeableness as well as Conscientiousness such that females have stronger correlations (see Table 3). Note that the Bonferroni correction for correlations coefficients reported in this table was calculated on the correlation matrix including correlations between the variables s-IAT, SPAI and personality scales.

**Table 3:** Common personality relationships to short internet addiction test (s-IAT)/smartphone addiction inventory (SPAI) by gender.

	**Present study**			**Original study**		
	**SPAI**	***t***	**s-IAT**	**SAS**	**Fisher's *z***	**IAT**
**MALES**						
Agreeableness	−0.01	−2.18^*^	−0.14	−0.13	*ns*	−0.19^*^
Conscientiousness	−0.01	−4.47^***^	−0.27^***^	−0.26^**^	1.9^*^	−0.44^***^
Extraversion	0.00	−3.68^***^	−0.22^**^	−0.09	*ns*	−0.08
Neuroticism	0.17^*^	4.50^***^	0.42^***^	0.35^***^	*ns*	0.39^***^
Openness	−0.13	1.81	−0.02	−0.12	−1.6^*^	0.06
**FEMALES**						
Agreeableness	−0.10	−3.15**	−0.23***	−0.11^*^	*ns*	−0.19^***^
Conscientiousness	−0.19^***^	−3.13^**^	−0.32^***^	−0.22^***^	*ns*	−0.27^***^
Extraversion	−0.01	−3.71^***^	−0.17^**^	0.05	2.6^**^	−0.13^**^
Neuroticism	0.21^***^	3.11^**^	0.33^***^	0.17^**^	*ns*	0.26^***^
Openness	−0.12^*^	3.35^**^	0.02	−0.14^**^	−2.5^**^	0.03

* *p < 0.05*,

** *p < 0.01*,

*** *p < 0.001, ns = not significant, Holm-Bonferroni corrected*.

### 3.4. BIS-15, IAS, and s-IAT/SPAI

BIS-15 and its subscales showed highly significant, positive correlations with both s-IAT and SPAI. IAS was positively correlated with both s-IAT and SPAI, the correlation with s-IAT being highly significant (see Table 4 for more details).

**Table 4:** Correlations between impulsivity (BIS-15), social anxiety (IAS) and short internet addiction test (s-IAT)/smartphone addiction inventory (SPAI).

	**s-IAT**	***t*_(770)_**	**SPAI**
BIS-15	0.38^***^	2.49^*^	0.31^***^
BIS-15 A	0.31^***^	0.79	0.29^***^
BIS-15 M	0.25^***^	3.13^**^	0.15^***^
BIS-15 NP	0.31^***^	1.42	0.27^***^
IAS	0.34^***^	7.47^***^	0.12^*^

* *p < 0.05*,

** *p < 0.01*,

*** *p < 0.001, Holm-Bonferroni corrected*.

Differences in correlation patterns between s-IAT and SPAI could be found for IAS, such that s-IAT scores showed a stronger, positive correlation with IAS scores as compared to the correlation between SPAI and IAS scores [*t*_(770)_ = 7.47, *p* < 0.001]. Another, weaker difference was found in correlational patterns of the subscale BIS-15 M with s-IAT and SPAI such that the correlation between BIS-15 M and s-IAT was higher than between BIS-15 M and SPAI on a 0.01 alpha level [*t*_(770)_ = 3.13, *p* = 0.002]. As a result, the overall BIS-15 score also showed a slightly stronger link to s-IAT than to SPAI [*t*_(770)_ = 2.49, *p* = 0.013]. IAS correlates significantly higher with s-IAT than with SPAI. The correlation pattern with BIS-15 and its subscales with regards to both direction and strength of association were very similar for s-IAT and SPAI, at least for the subscales BIS-15 A and BIS-15 NP. Except for the subscale BIS-15 M, BIS-15 correlated in a similar way with both s-IAT and SPAI.

**Figure 2:**
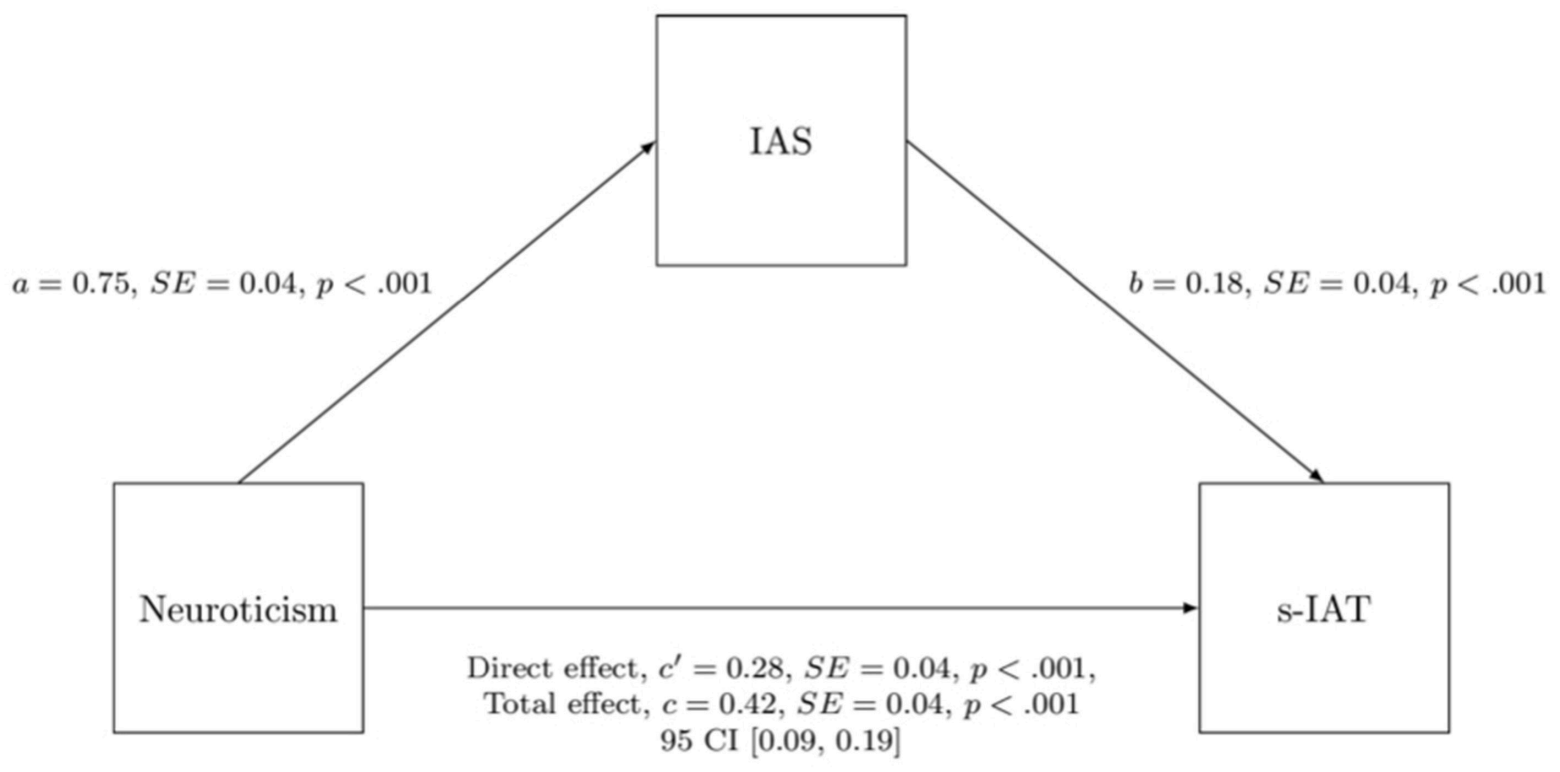
IAS functions as a mediator between Neuroticism and s-IAT.

For completeness, we also report associations between personality and impulsivity (BIS-15) and social anxiety (IAS) as depicted in Table 5. Because correlations between Neuroticism and s-IAT, IAS and s-IAT as well as between Neuroticism and IAS were all very high, we conducted a path analysis to determine whether IAS acts as a mediator with respect to the association between Neuroticism and s-IAT. The analysis revealed that IAS acts as a partial mediator in the association between Neuroticism and s-IAT (see Figure 2). IAS did not mediate the link between Neuroticism and SPAI. In fact, the total and direct effects were very similar, so removing IAS did not make a difference in the association between Neuroticism and SPAI (*c* = 0.45, *c*′ = 0.43).

**Table 5:** Correlations between impulsivity (BIS-15), social anxiety (IAS) and the big five of personality.

	**BIS-15**	**BIS-15 A**	**BIS-15 M**	**BIS-15 NP**	**IAS**
Agreeableness	−0.26^***^	−0.19^***^	−0.23^***^	−0.19^***^	−0.22^***^
Conscientiousness	−0.51^***^	−0.32^***^	−0.57^***^	−0.31^***^	−0.26^***^
Extraversion	−0.15^***^	−0.16^***^	−0.22^***^	0.02	−0.73^***^
Neuroticism	0.48^***^	0.40^***^	0.34^***^	0.39^***^	0.54^***^
Openness	−0.02	−0.10	−0.02	0.10	0.12^*^

* *p < 0.05*,

*** *p < 0.001, Holm-Bonferroni corrected*.

### 3.5. Present Study vs. Original Study

The correlation patterns between personality variables and s-IAT/SPAI scores were very similar to the correlation patterns found in the original study between personality variables and IAT/SAS scores, respectively. In fact, the only significant differences could be found in the correlation between Neuroticism and IAT/s-IAT and in the correlation between Conscientiousness and SPAI/SAS. In the present study, the positive correlation between Neuroticism and s-IAT was higher than the correlation between Neuroticism and IAT in the original study (Neuroticism and IAT: *r*_*s*_ = 0.26, Neuroticism and s-IAT: *r*_*s*_ = 0.35, *z* = 2.03, *p* = 0.042), whereas Conscientiousness was significantly more negatively correlated with SAS than with SPAI (Conscientiousness and SAS: *r*_*s*_ = −0.23, Conscientiousness and SPAI: *r*_*s*_ = −0.10, *z* 2.57, *p* = 0.010). All other Fisher's *z*-tests performed to compare overall correlation patterns between the original study and present study were not significant. Note also that a different Big Five measure was used in the present study compared to the work by Lachmann et al. (1).

## 4. Discussion

The main goal of this work was to replicate findings from the article by Lachmann et al. (1) using other questionnaires to assess IUD, SUD as well as personality. Finding similar associations between SUD/IUD and personality traits in a different sample from different countries and using different questionnaires would support the hypothesis that findings are robust regardless of nationalities and measurement method. We expected to find both high IUD and SUD scores to be associated with high Neuroticism, low Conscientiousness as well as low Agreeableness. We further expected a negative correlation between Extraversion and IUD and between Openness and SUD but no correlation between Extraversion and SUD nor Openness and IUD. Correlation patterns should be similar for both genders [according to (1)] and a common underlying trait for IUD and SUD should be found, reflecting the underlying, common personality structure. In fact, most findings were replicated which suggests that results of the work by Lachmann et al. (1) can be considered generally valid. This said, future work might also aim at conducting the present research in Asian countries to assess if similar results can be observed when Eastern cultures are investigated. Beyond replication of the study by Lachmann et al. (1), we further attempted to replicate findings of earlier studies showing that high social anxiety and high impulsivity are both associated with IUD and SUD (50, 52, 55, 56).

In accordance with the I-PACE model and/or the original study, high IUD and SUD scores showed associations with high Neuroticism, low Conscientiousness and low Agreeableness (note that the Agreeableness-SUD, Conscientiousness-SUD links were not significant), while Extraversion was negatively correlated with IUD but not correlated with SUD, and Openness was negatively correlated with SUD but not correlated with IUD. In particular high Neuroticism and to a lesser extent lower Conscientiousness represent the common personality structure underlying IUD and SUD in this work, too. In addition, IUD had significantly stronger correlations with Neuroticism, Extraversion, Agreeableness and Conscientiousness than SUD. SUD on the other hand had a stronger association with Openness than IUD. The same difference in strength of association was also observed in the study by Lachmann et al. (1), except for Neuroticism (note: the difference was not significant in the original study). Please note that future works should also take more into account the variable of gender in the context of personality-SUD associations, because in the present work in particular the inverse Conscientiousness-SUD correlation could only be observed in the female subgroup. This is a difference compared to the Lachmann et al. (1) study where higher SUD scores were associated with lower Conscientiousness scores in both males and females.

Besides the underlying, common personality structure and differences between IUD and SUD which all were largely similar to the original study, we also found that even the strength of association between IUD/SUD and the single investigated personality traits was comparable across studies. One difference compared with the original study however can be found regarding Neuroticism and Conscientiousness. In fact, in the present sample Neuroticism showed a significantly higher, positive correlation to IUD than in the original work whereas Conscientiousness showed a significantly lower, negative correlation to SUD than in the original study. One possible explanation could lie in the difference of administered self-report instruments. Of note, in the case of Conscientiousness, the observed correlation can be narrowed down to the SPAI subscale “Functional Impairment” in the present work (overall: rs = − 0.15, p < 0.001; males: rs = − 0.10, p = 0.098; females: rs = − 0.21, p < 0.001). Items belonging to that subscale are for example “I feel aches and soreness in the back or eye discomfort due to excessive smartphone use” and “I feel tired on daytime due to late-night use of smartphone”. Because very similar items can be found in the SAS (“Feeling pain in the wrists or at the back of the neck while using a smartphone” and “Feeling tired and lacking adequate sleep due to excessive smartphone use”), we would rule out the possibility that the lower, negative correlation between Conscientiousness and SPAI is due to a difference in the way SPAI (rather than SAS) measures SUD. Another possible explanation could be that differences appear because different instruments were used to measure personality. In fact, Conscientiousness is measured slightly differently in the NEO-FFI as compared with the TSDI. Last but not least, it is also conceivable that differences are due to the samples being different from one another in regard to their Conscientiousness scores. More research needs to address this question. Of note, the weaker association between Conscientiousness and SUD and a bit stronger association between Neuroticism and IUD found in the present work do not alter the validity of the conclusions of Lachmann et al. (1), but rather reinforces them. Interestingly, in the case of Neuroticism and its correlation with s-IAT, we found that IAS partially mediates the association which is not true for the correlation between Neuroticism and SPAI. Thus, the mediation effect of IAS between Neuroticism and s-IAT represents a further difference in the underlying personality structures of IUD and SUD.

Beyond the Big Five personality associations, we expected impulsivity and social anxiety to be positively associated with IUD and SUD because both have been pointed out as risk factors in the I-PACE model (P component). In line with our expectations based on the model and past research (33), we found that s-IAT and SPAI were positively correlated with overall BIS-15 scores as well as with the subscales motor impulsivity (BIS-15 M), non-planning impulsivity (BIS-15 NP) and attentional impulsivity (BIS-15 A). Participants with high s-IAT and/or SPAI score were also more likely to have high BIS-15 scores. The only difference in correlational patterns between BIS-15 and s-IAT/SPAI could be found with motor impulsivity (BIS-15 M), such that the link between BIS-15 M and s-IAT was significantly stronger than the link between BIS-15 M and SPAI. Because the other subscales had very similar links to both s-IAT and SPAI, it is safe to conclude that overall, the correlational pattern with BIS-15 is similar for both s-IAT and SPAI. In general, the associations with impulsivity are meaningful, as addictive behavior often reflects in impulsive drug use, hence the association is not surprising and is in line with much conducted research related to addictive behaviors, as also cited in the introduction. With respect to social anxiety, results are in line with past research showing that individuals with high scores in social anxiety scales also had a higher tendency toward IUD and/or SUD scores (55, 56). Interestingly, the correlation between s-IAT and IAS was significantly stronger than the correlation between SPAI and IAS (the latter only reached significance using alpha of 0.05). One possible explanation could be that social anxiety does not affect one's likelihood to use a smartphone as much as it affects one's likelihood to use the Internet in general. Socially anxious individuals may spend more time in front of a desktop computer avoiding face to face encounters in real life and engaging in detrimental Internet activities more than non-socially anxious individuals who may spend less time in front of their desktop computers and engage more with peers in real-life.

The results of this study are subject to certain limitations. In fact, participants showed little variance in age and were mostly in their twenties which limits generalizability of the research to older cohorts. Furthermore, participants were recruited from several different cultural backgrounds, sometimes represented by just few participants, potentially masking the effect of culture. The fact that removing participants from non-English speaking countries did not seem to impact the results could thus be due to underrepresentation of other cultures in our sample. Further research should investigate the effect of nationality, especially taking countries with cultural backgrounds that are different from Germany and the USA into consideration. Finally, the present work is of correlational nature. Therefore, no causality can be inferred from this study's results and longitudinal work is warranted to understand causal links between the investigated variables.

## 5. Supplement

In this section, we report the results of the hierarchical regression analysis predicting s-IAT and SPAI. The regressions were conducted in three blocks to outline the predictive power of the different variables. The first block consisted of age and gender as independent variables because they have shown relationships with IUD (in the original study) and SUD (in the original and the present study). In the second block, the five personality variables were added as independent variables to the models. In the third block, impulsivity and social anxiety were added as the final variables to the models. The explained variances reported below are adjusted *R*^2^ values.

### 5.1. Regression Anaylsis IAT

(Low) Age, Gender (male), (high) Extraversion, (low) Conscientiousness, (high) Neuroticism, (high) IAS as well as (high) BIS-15 are the significant predictors of s-IAT scores (see Table 6). Demographic variables alone explained 5.29% of the variance while Big Five variables added a further 16.58% to the model, resulting in a total of 21.87% of variance explained by the second model. The model that accounted for the most variance was the third model with 26.17% of variance explained [*F*_(763)_ = 31.41, *p* < 0.001]: Adding IAS and BIS-15 scores to the equation yielded an additional 4.30% of explained variance. Unlike in the correlation analysis, (low) Agreebleness did not reach significance. Besides this difference, the results are in line with those of the correlation analysis. The predictive power of single variables changes somewhat when adding variables to the model, but not substantially.

**Table 6:** Summary of hierarchical regression analysis for variables predicting s-IAT (*N* = 733).

	**Model 1**			**Model 2**			**Model 3**		
	**B**	**SE B**	**β**	**B**	**SE B**	**β**	**B**	**SE B**	**β**
Intercept	29.23	1.12	26.02^***^	28.28	3.14	9.01^***^	7.66	4.31	1.77
Age	−0.30	0.05	−6.55^***^	−0.26	0.04	−6.16^***^	−0.22	0.04	−5.26^***^
Gender	1.03	0.68	1.52	1.60	0.64	2.49*	1.52	0.62	2.43^*^
Extraversion				−0.03	0.05	−0.60	0.16	0.06	2.45^*^
Conscientiousness				−0.28	0.06	−4.37^***^	−0.18	0.07	−2.56^*^
Openness				−0.03	0.05	−0.52	−0.01	0.05	−0.19
Agreeableness				−0.10	0.05	−1.95	−0.08	0.05	−1.51
Neuroticism				0.34	0.04	8.09^***^	0.17	0.05	3.43^***^
IAS							0.21	0.04	4.97^***^
BIS-15							0.20	0.05	4.16^***^

* *p < 0.05*,

*** *p < 0.001*.

### 5.2. Regression Analysis SPAI

(Low) Age, Gender (female), (low) Openness, (high) Neuroticism and (high) BIS-15 are the significant predictors of SPAI scores (see Table 7). In this regression anaylsis, demographic variables alone explained 5.23% of the variance while Big Five variables explained an additional 7.17% of SPAI scores, which yields a total of 12.40% of variance explained by the second model. The third model explains 16.58% of the variance [*F*_(763)_ = 18.05, *p* < 0.001). Adding IAS and BIS-15 scores to the equation yielded an additional 4.18% of explained variance, although the IAS score was not a significant predictor. The results of the regression analysis correspond to the trends uncovered in the correlation analysis of the main section. The predictive power of single variables remains almost unchanged when adding variables to the model.

**Table 7:** Summary of hierarchical regression analysis for variables predicting SPAI (*N* = 733).

	**Model 1**			**Model 2**			**Model 3**		
	**B**	**SE B**	**β**	**B**	**SE B**	**β**	**B**	**SE B**	**β**
Intercept	58.02	1.88	30.90^***^	55.09	5.56	9.91^***^	30.06	7.67	3.92^***^
Age	−0.45	0.08	−5.97^***^	−0.36	0.07	−4.86^***^	−0.34	0.07	−4.66^***^
Gender	−3.34	1.13	−2.95^**^	−2.25	1.14	−1.99^*^	−2.49	1.11	−2.25^*^
Extraversion				0.17	0.08	2.01^*^	0.18	0.11	1.56
Conscientiousness				−0.21	0.11	−1.87	0.10	0.12	0.79
Openness				−0.43	0.09	−4.94^***^	−0.35	0.09	−4.12^***^
Agreeableness				−0.01	0.09	−0.14	0.04	0.09	0.43
Neuroticism				0.48	0.08	6.41^***^	0.23	0.09	2.60^**^
IAS							0.09	0.08	1.21
BIS-15							0.52	0.09	6.09^***^

* *p < 0.05*,

** *p < 0.01*,

*** *p < 0.001*.

## Ethics Statement

The study has been approved by the local ethic committee of Ulm University, Ulm, Germany. Participants gave electronic consent.

## Author Contributions

The study was designed by JP-B and CM. Data collection has been carried out by JP-B and CM. Statistical analyses have been conducted by JP-B, whereas CM advised on some of the statistical approaches. The manuscript has been written by JP-B, CM, and JE. In detail JP-B drafted the complete method and statistics section, whereas JP-B, CM, and JE together drafted the introduction. JP-B drafted the discussion which then was critically revised by CM and JE. CS independently checked all statistical results. The final version has been approved by all authors.

### Conflict of Interest Statement

The authors declare that the research was conducted in the absence of any commercial or financial relationships that could be construed as a potential conflict of interest.
